# Irisin Improves Myocardial Performance and Attenuates Insulin Resistance in Spontaneous Mutation (*Lepr^db^*) Mice

**DOI:** 10.3389/fphar.2020.00769

**Published:** 2020-06-03

**Authors:** Jianguo Wang, Yu Tina Zhao, Ling Zhang, Patrycja M. Dubielecka, Shougang Zhuang, Gangjian Qin, Yu Eugene Chin, Shouyan Zhang, Ting C. Zhao

**Affiliations:** ^1^Department of Surgery, Boston University School of Medicine, Roger Williams Medical Center, Providence, RI, United States; ^2^University of Rochester School of Medicine and Dentistry, Rochester, NY, United States; ^3^Department of Medicine, Rhode Island Hospital, Brown University, Providence, RI, United States; ^4^Department of Biomedical Engineering, University of Alabama at Birmingham, Birmingham, AL, United States; ^5^Translation Medicine Center, Shanghai Chest Hospital, Shanghai Jiao Tong University, Shanghai, China; ^6^Department of Medicine, Luoyang Central Hospital, Zhengzhuo University, Luoyang, China; ^7^Department of Surgery, Rhode Island Hospital, Brown University, Providence, RI, United States

**Keywords:** irisin, diabetes, myocardial function, insulin resistance, p38 and HDAC4

## Abstract

**Background:**

Irisin, a newly identified peptide, is critical to regulating metabolism, thermogenesis, and reducing oxidative stresses. Our recent works demonstrated that irisin protected the heart against myocardial ischemic injury and preserved the function of mitochondria. However, whether irisin preserves myocardial performance and attenuates insulin resistance in type II diabetes remains unknown.

**Objective:**

Effects of irisin on type II diabetes-induced cardiac dysfty unction and insulin resistance in db/db mice were studied. Methods: Homozygous db/db mice (n=5/each group) for spontaneous mutation (*Lepr^db^*) and heterozygous (heterozygous) mice (n=5/each group) for control were used to assess for cardiac performance and impairment of insulin resistance. Homozygous and heterozygous controls received a treatment with either irisin (100 mg/kg, intraperitoneal injection, every other day) or vehicle control (PBS) for 4 weeks at 16 weeks of age. Insulin tolerance test and glucose tolerance test were employed to determine insulin resistance in mice. Cardiac function was assessed by echocardiography. Metabolic features including hyperglycemia and body growth were also examined. Immunohistochemical analysis was employed to determine myocardial hypertrophy and interstitial fibrosis. Immunoblots were employed to determine the signaling pathway associated with irisin treatment.

**Results:**

Homozygous db/db mice developed an impairment in insulin sensitivity as indicated by Insulin tolerance test (ITT), glucose tolerance test (GTT) (p<0.05 vs non-irisin treatment), hyperglycemia (p<0.05 vs heterozygous control), and hyperinsulinemia (serum insulin: 0.81 ± 0.065 ng/ml in heterozygous control vs. 8.33 ± 0.69 ng/ml in homozygous db/db control, p<0.0001), which were attenuated by the administration of irisin (serum insulin 8.32 ± 0.68 ng/ml in homozygous db/db control vs 6.56 ± 0.38 ng/ml in homozygous db/db irisin treatment, p<0.0001). Furthermore, as compared to heterozygous control, db/db mice manifested a depression in cardiac performance [ejection fraction (EF): 91.9% ± 0.44 in heterozygous control vs 79.1% ± 2.0 in homozygous db/db control, p< 0.001] in associated myocardial remodeling (cardiac fibrosis 1.89% ± 0.09 in heterozygous control vs. 5.39% ± 0.22 in homozygous db/db control, p<0.001). Notably, the depression of cardiac function in EF (79.2% ± 2.0 homozygous db/db control vs. 88.6% ± 1.9 in homozygous db/db + irisin, p<0.01) and fractional shortening (FS) (42.2% ± 1.8 in homozygous db/db control vs. 53.2% ± 2.7 in homozygous db/db+irisin, p<0.01) and remodeling were markedly attenuated by the administration of irisin. Western blotting shows that irisin treatment prevented an approximate two-fold decrease in p38 phosphorylation and increase in histone deacetylase 4 (HDAC4) in the homozygous db/db myocardium (p<0.05 vs homozygous db/db control).

**Conclusion:**

Irisin preserves myocardial performance and insulin resistance in db/db mice, which is related to p38 phosphorylation and HDAC reduction.

## Background

Recent statistics indicate an inexorable increase in the prevalence of diabetes, obesity, and insulin resistance in the developed world and emerging economies (Abel, 2010). Diabetes mellitus is associated with increased (Kannel et al., 1974) cardiovascular disease, which is considered to be the major cause of morbidity and mortality. Cardiomyopathy develops in diabetic disease that is independent of known risk factors such as hypertension as well as coronary artery disease (Rubler et al., 1972). It is commonly understood that the underlying mechanisms of diabetes-related heart disease are multifactorial, but the importance of studying myocardial dysfunction, termed as *diabetic cardiomyopathy*, has always been a focus in the field (Witteles and Fowler, 2008; Battiprolu et al., 2010). Irisin has been recently identified as proliferator-activated receptor-gamma coactivator-1α (PGCγ-1α)-dependent myokine. Irisin is secreted by muscle cells and subsequently circulated into peripheral tissue when exercise as a cleavage peptide of the extracellular portion of type I membrane protein fibronectin type III domain containing 5 (FNDC5) (Boström et al., 2012). It was recently considered to act as a kind of hormone that produces the beneficial function of exercise on promoting the browning of white adipose tissues and stimulating energy expenditure (Boström et al., 2012). It has also been shown to suppress oxidative stresses and apoptosis in different models (Zhu et al., 2015; Park et al., 2015). It was reported that irisin augmented the browning of white adipose tissue, which could serve as a promising therapy for metabolic disorders and cardiovascular diseases (Jeremic et al., 2017). Very recently, we have demonstrated that pretreatment of irisin protected the heart against ischemia/reperfusion injury and attenuated the susceptibility of cardiomyoblasts exposed to hypoxic stress, which is closely related to the improvement in mitochondria respiration, reduction of mitochondria apoptosis, inhibition of histone deacetylase 4 (HDAC4) (Zhang et al., 2014; Zhao et al., 2016; Wang et al., 2017). In addition, our recent studies and others also indicate that irisin suppressed metabolic stress in cell models and induced protection were associated with the activation of AMP-activated protein kinase (AMPK), insulin signaling pathway and stimulation of p38 mitogen-activated protein (MAP) kinase (Zhang et al., 2014; Li et al., 2019). It was found that irisin protected against endothelial injury and ameliorated atherosclerosis in apoE ^-/-^ diabetic mice, suggesting that irisin is considered as an approach for therapy for atherosclerotic vascular diseases in diabetes (Lu et al., 2015). Also, a decreased peripheral and vitreous irisin levels were found in type 2 diabetes patients (Hu et al., 2016). Irisin was shown to be abundantly presented in the muscle, pituitary of adult myocardium (Li et al., 2015). Irisin also regulated the neurons in the control of the cardiac vagal tone by modulating central cardiovascular regulation (Brailoiu et al., 2015). It is important to determine whether irisin could generate a protective effect against cardiac dysfunction and remodeling in diabetic model, which could be developed as a novel treatment for diabetic cardiomyopathy and metabolic disease.

In this study, we employed an homozygous db/db and heterozygous mice *in vivo* to determine 1) if irisin treatment could increase the resistance of the mice to development of myocardial dysfunction and remodeling; 2) Whether irisin treatment prevents cardiac remodeling and attenuate insulin resistance; 3)Whether irisin-induced physiological function is associated with p38 mitogen-activated protein kinase (MAPK) and HDAC4 in db/db mice. Our findings suggest that irisin produced a protective effect against development of cardiac dysfunction in db/db mice as demonstrated by the preserving cardiac function and reducing interstitial fibrosis in association with the suppression of insulin intolerance, which is related to p38 phosphorylation and attenuated HDAC4

## Methods and Materials

### Animals

Four-week old homozygous db/db mice for spontaneous mutation (*Lepr^db^*) and heterozygous male mice for control were obtained from Jackson Laboratory (Bar Harbor, Maine), respectively. Animals were housed in accordance with standard animal care requirements and maintained on a 12/12 h light-dark cycle in Central Animal Facility. Water and food were given *ad libitum*. The studies for all animal experiments were conducted according to a protocol approved by the Institutional Animal Care and Use Committee of Roger Williams Medical Center, which conforms to the Guide for the Care and Use of Laboratory Animals published by the US National Institutes of Health (NIH Publication No. 85-23, revised 1996).

### Reagents and Antibodies

Recombinant mouse irisin was purchased from Cayman Chemical (Michigan, USA). All other chemicals were obtained from Sigma-Aldrich (St. Louis, MO). Primary antibodies including polyclonal wheat germ agglutinin (WGA, FITC-conjugated) was obtained from Sigma-Aldrich (St. Louis, MO). Goat anti-Rat secondary antibody (Alexa Fluor^®^ 555 conjugate) was purchased from (Life Technologies, Carlsbad, CA). Human insulin was obtained from the pharmacy of Roger William Medical Center (Providence, RI).

### Experimental Protocol and Irisin Treatment

**Figure 1:**
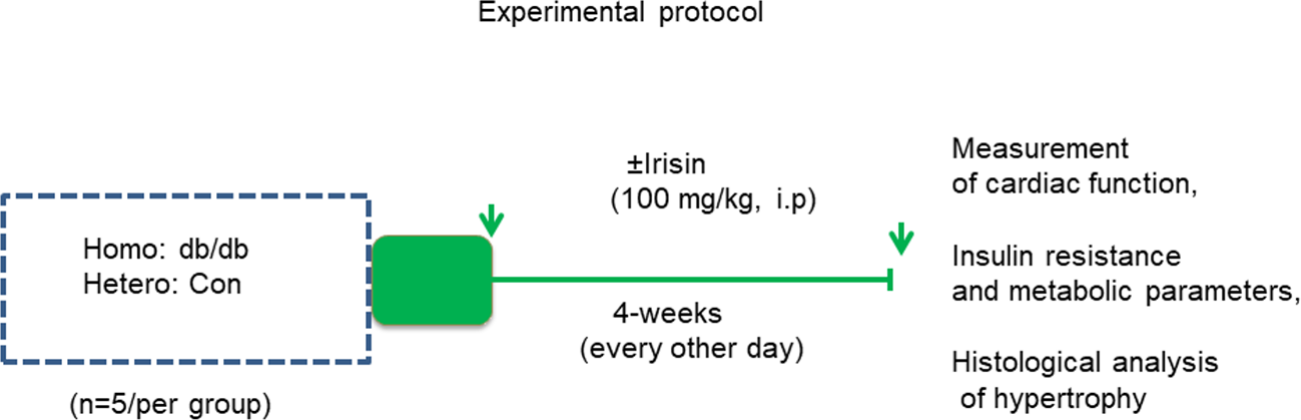
Schemas of animal experimentation in homozygous db/db mice for spontaneous mutation (*Lepr^db^*) and heterozygous mice. Echo: echocardiography; Detailed descriptions of the experiments are listed in the section of methods (heterozygous control=5; heterozygous+irisin=5; homozygous db/db control=5. homozygous db/db+irisin=5); Hetero, heterozygous mice; Homo, homozygous mice.

In order to assess the effect of irisin on attenuating the progressive cardiac dysfunction in homozygous mice, both homozygous db/db mice for spontaneous mutation (*Lepr^db^*) and heterozygous mice for control were subjected to receiving either irisin treatment or vehicle. As shown in **Figure 1**, both homozygous and heterozygous controls received a treatment with either irisin at a dose of 100 mg/kg *via* intraperitoneal injection every other day or vehicle control (PBS) for 4 weeks at sixteen-week old of age. The dose of irisin treatment was based on our previous study in which irisin showed a protective effect against myocardial ischemia and reperfusion injury (Wang et al., 2017). Assessment of cardiac function and metabolic parameters were conducted in the course of experiment.

### The Measurement of the Glucose Tolerance Tests (GTT) and Insulin Resistance Test (ITT)

Homozygous db/db mice for spontaneous mutation (*Lepr^db^*) and heterozygous mice for control were fasted for 4 h, and then baseline glucose concentrations were assessed using an Accu-Chek Compact Plus glucometer (Roche Diagnostics, Indianapolis, IN) from tail blood. Mice were then injected (intraperitoneally, i.p) with 1 mg/g glucose. Blood glucose was determined at 15, 30, 60, 120, and 240 min following i.p. injection of glucose. Total serum fasting glucose from tail blood were also detected with a CardioChek PA system (PTS Diagnostics, Indianapolis, IN) under the manufacturer’s instructions. Detailed methodology was described as the previous protocol with a minor modification (Chen et al., 2015; Zhang et al., 2017). Insulin level in serum was determined using a standard curve obtained from the standards provided by the kit. Serum insulin was assessed using a Triglyceride Colorimetric Assay Kit from Cayman Chemical (Ann Arbor, MI) according to the manufacturer’s instructions (Zhang et al., 2017).

### Cardiac Functional Measurements

Echocardiographic measurement of cardiac function was performed by using an Acuson Sequoia C512 system equipped with a 15L8 linear array transducer. Mice were anesthetized with isoflurane (5%) that was mixed with oxygen through a nose cone, and then placed in supine position on a warming pad to maintain boy temperature. The precordial region was applied with ultrasound transmission gel (Aquasonic, Parker Laboratory, Fairfield, NJ) following air removal. Short axis measurements were employed to capture two-dimensional B-mode images and M-mode tracing at the level of the papillary muscles. The measurement was taken at 25 mm signal depth. Three to six consecutive cardiac cycles were collected from the measurement of M-mode tracings on accompanying software. All measurements were conducted by an experienced operator blinded to mouse strains and treatments (Zhang et al., 2014; Du et al., 2016).

### Immunohistochemistry

The protocol of immune histological analysis was conducted with the minor modification as described briefly. Paraffin-embedded tissues were used to determine cell surface area. De-paraffin slides were probed with wheat germ agglutinin (WGA, FITC-conjugated) at 4°C, and then was counterstained with 4′, 6-Diamidino-2-phenylindole (DAPI) to stain nuclei. at room temperature for 1 h. Images was acquired using a Carl Zeiss LSM 700 laser scanning microscope equipped with intuitive ZEN 2009 software. Five to 10 randomly selected fields were selected for quantifications using NIH ImageJ software (NIH, version 1.40 g, http://rsb.info.nih.gov/ij/). To evaluate interstitial myocardial fibrosis, left ventricular sections were stained with picrosirius red that was quantitated in images obtained using a microscope coupled to a computerized morphometry system (Olympus BX41). Myocardial collagen content was expressed as a percentage of cardiac area in the image. The size of white fat tissues was also determined using standard H&E staining. The cross-sectional areas of adipocyte white cells were measured from 200 to 250 sample size (Zhang et al., 2012).

### Western Blotting

Protein contents were measured by western blotting using tissue lysates (50 μg/lane) as described previously (Zhao et al., 2016). β–actin protein was included as the loading control. Immunoblots were visualized with the chemiluminescence reagent (Super Signal West Pico ECL, Thermo-Fisher Scientific). Polyclonal rabbit phospho-p38, total p38, and HDAC4 primary antibodies (Cell Signaling Technology) at the concentration (1:1,000) were probed to assess the protein levels of myocardium, respectively. Densitometric analysis for the blots were done using NIH Image J processing program.

### Statistical Analysis

The results are expressed as means ± SE. Difference among the groups were analyzed by two-way analysis of variance (ANOVA), followed by *post hoc* Bonferroni correction. Statistical differences were considered to be significant with a value of p<0.05.

## Results

### Irisin Attenuates Hypercholesterolemia and Obesity in Homozygous db/db Mice for Spontaneous Mutation (*Lepr^db^*)

**Figure 2:**
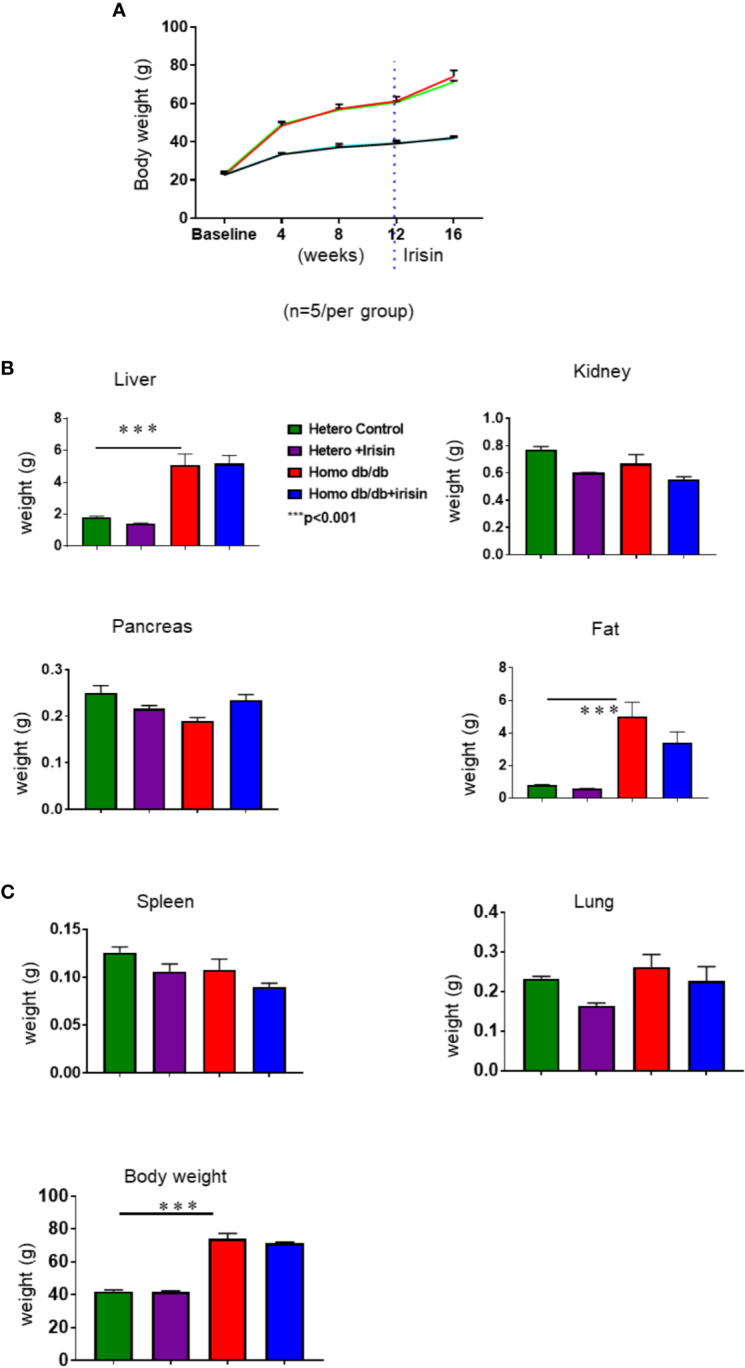
Irisin treatment on body weight in spontaneous mutation (*Lepr^db^*) obese mice. **(A):** Body weights in the course of 4 week of irisin treatment; **(B, C):** Individual organ change in homozygous db/db mice and heterozygous mice at the end of experiments. Data are shown as means ± SEM. (heterozygous control=5; heterozygous+irisin=5; homozygous db/db control=5. homozygous db/db+irisin=5), ***p < 0.001 vs heterozygous control and heterozygous+irisin.

Homozygous db/db mice for spontaneous mutation (*Lepr^db^*) and heterozygous mice for control were exposed to standard food conditions. As shown in **Figure 2A**, the body weight of mice was significantly increased in the homozygous db/db after 4 weeks, as compared to heterozygous mice. This phenotypic change in body weight from homozygous db/db indicates the development of obesity (**Figure 2A**). However, the increase in the body weights of homozygous db/db group was slightly prevented by treatment of the mice with irisin. As shown in **Figures 2B, C**, individual organs including liver, kidney also demonstrated the increase in homozygous db/db mice, but the magnitude of these increases was also partially prevented by administration of irisin. Notably, the subcutaneous adipose was remarkably reduced by administration of irisin.

### Irisin Restores Induced Left Ventricular Dysfunction in Homozygous db/db Mice

**Figure 3:**
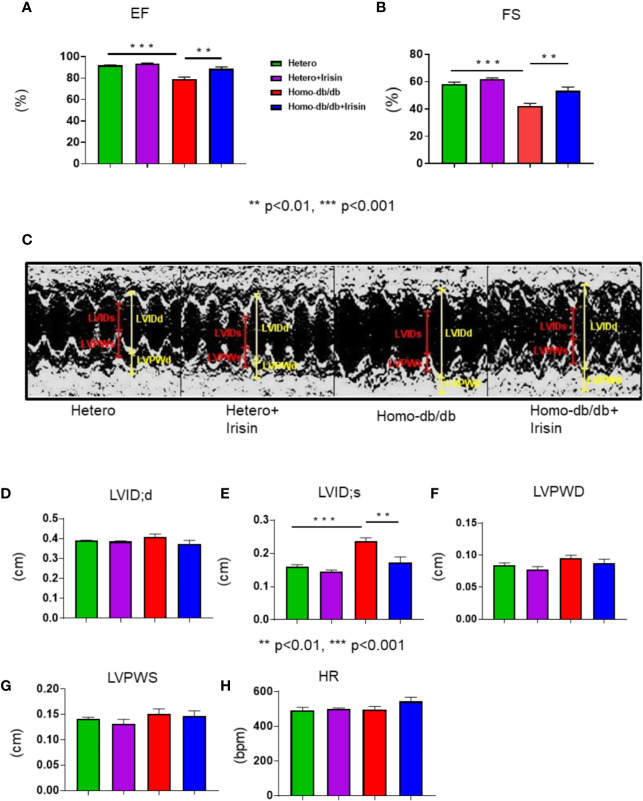
Irisin treatment improved impaired left ventricular function in homozygous db/db mice for spontaneous mutation (*Lepr^db^*) mice. Echocardiographic measurements of ventricular functional parameters includes **(A)**: Fractional Shortening (FS) **(B)**: Ejection Fraction (EF); **(C)**: Representative image of M-mode; **(D)**. Left Ventricular Internal Dimension in Diastole (LVID;d); **(E)**: Left Ventricular Internal Dimension in Systole (LVID;s); **(F, G)**: Left ventricular posterior wall (LVPW;s and LVPW;d); **(H)**: heart rate (HR). Data are shown as means ± SEM (heterozygous control=5; heterozygous+irisin=5; homozygous db/db control=5. homozygous db/db+irisin=5). **p < 0.01, ***p < 0.001.

Echocardiographic assessment was performed to measure LV function in tested mice. As shown in **Figures 3A–C**, homozygous db/db mice exhibited a depression in myocardial function, as indicated by the dramatic reduction in fractional shortening (FS) (58.2%± 1.4 in heterozygous control vs 42.4% ± 1.8 in homozygous db/db control) and ejection fraction (EF) (91.9% ± 0.4 vs 79.2% ± 2.0 in homozygous db/db control as compared with heterozygous mice, p<0.001). Likewise, homozygous db/db mice also displayed an increased left ventricular internal dimension (LVID) in systolic and diastolic stages versus heterozygous mice (**Figures 3D, E**) (LVID:s, 0.16 ± 0.001 mm in heterozygous control vs.0.24 ± 0.01 mm in homozygous db/db control p<0.001). However, the cardiac dysfunction in EF (79.2% ± 2.0 homozygous db/db control vs. 88.6% ± 1.9 in homozygous db/db+irisin, p<0.01), FS (42.2% ± 1.8 in homozygous db/db control vs. 53.2% ± 2.7 in homo db/db+irisin, p<0.01), and LVID:s, (0.24 ± 0.01 mm in homo db/db control vs 0.17 ± 0.02 mm in homo db/db+irisin, p<0.01) in homozygous db/db mice was prevented by the administration of irisin. Wall thickness (LVPWD) slightly decreased in homozygous db/db mice following irisin treatment (**Figures 3F, G****)** (0.095 ± 0.004 mm in homozygous db/db control vs 0.087 ± 0.006 mm in homozygous db/db +irisin, p>0.05). In addition, the heart rates were not affected by administration of irisin among homozygous db/db and heterozygous mice.

### Irisin Attenuates Homozygous db/db Induced Glucose Intolerance

**Figure 4:**
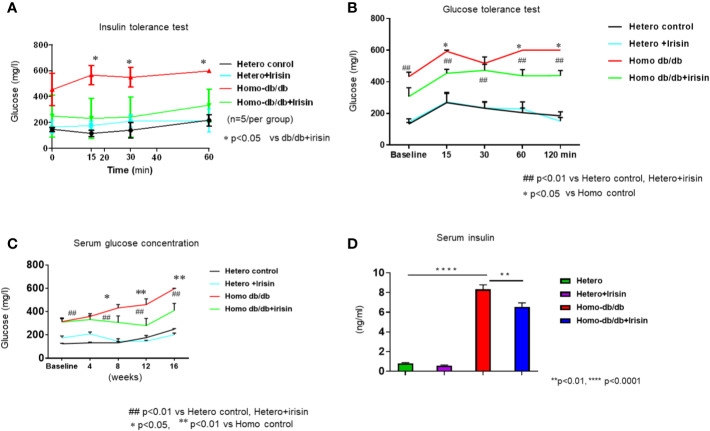
Irisin treatment attenuates insulin resistance in homozygous db/db mice for spontaneous mutation (*Lepr^db^*) obese mice. **(A)**: Insulin tolerance testhomozygous db/db mice for spontaneous mutation (*Lepr^db^*) and heterozygous mice after 16 weeks. **(B):** Glucose tolerance test in homozygous db/db mice for spontaneous mutation (*Lepr^db^*) and heterozygous mice after 16 weeks. **(C)**: Irisin attenuating hyperglycemia in homozygous db/db mice. *p < 0.05, **p < 0.01 vs vehicle treatment of homozygous db/db mice (n=5/per group). ^##^p < 0.01 vs Hetero control or Hetero+irisin. **(D)**: Irisin attenuating hyperinsulinemia in homozygous db/db mice. **p < 0.01, ****p < 0.0001 (heterozygous control=5; heterozygous+irisin=5; homozygous db/db control=5. homozygous db/db+irisin=4).

To determine whether irisin could mitigate whole body insulin sensitivity related to the development of metabolic disorder in homozygous db/db mice, insulin tolerance test was performed. As shown in **Figure 4A**, heterozygous mice displayed a normal glucose response as shown by insulin tolerance tests, as indicated by ITT, while homozygous db/db mice displayed insulin intolerance (p<0.05 vs heterozygous control at 15, 30, and 60 (min) of time course. Irisin corrected the insulin intolerance in homozygous db/db mice (p<0.05 vs homozygous db/db control). In addition, the glucose tolerance test was employed to assess insulin sensitivity, as shown in **Figure 4B**. Homozygous db/db mice displayed a severe glucose intolerance as compared to heterozygous control mice (p<0.01 vs heterozygous control at 15, 30, 60, and 120 min of time course). However, treatment of homozygous db/db mice with irisin attenuated glucose intolerance as compared with the control treatment (p<0.05 vs homozygous db/db control at 15, 60, and 120 min of time course). As shown in **Figure 4C**, hyperglycemia was manifested in homozygous db/db animals. Irisin treatment caused a dramatic decrease in hyperglycemia in homozygous db/db mice (p<0.05 vs homozygous db/db control). Likewise, as shown in **Figure 4D**, hyperinsulinemia was also exhibited in homozygous db/db animals as compared to heterozygous mice (serum insulin: 0.81 ± 0.065 ng/ml in heterozygous control vs. 8.33 ± 0.69 ng/ml in homozygous db/db control, p<0.0001), but irisin treatment also resulted in a marked decrease in serum insulin (serum insulin 8.32 ± 0.68 ng/ml in homozygous db/db control vs 6.56 ± 0.38 ng/ml in homozygous db/db irisin treatment, p<0.01 vs homozygous db/db control).

### Irisin Attenuated Cardiac Hypertrophy in Homozygous db/db Mice

**Figure 5:**
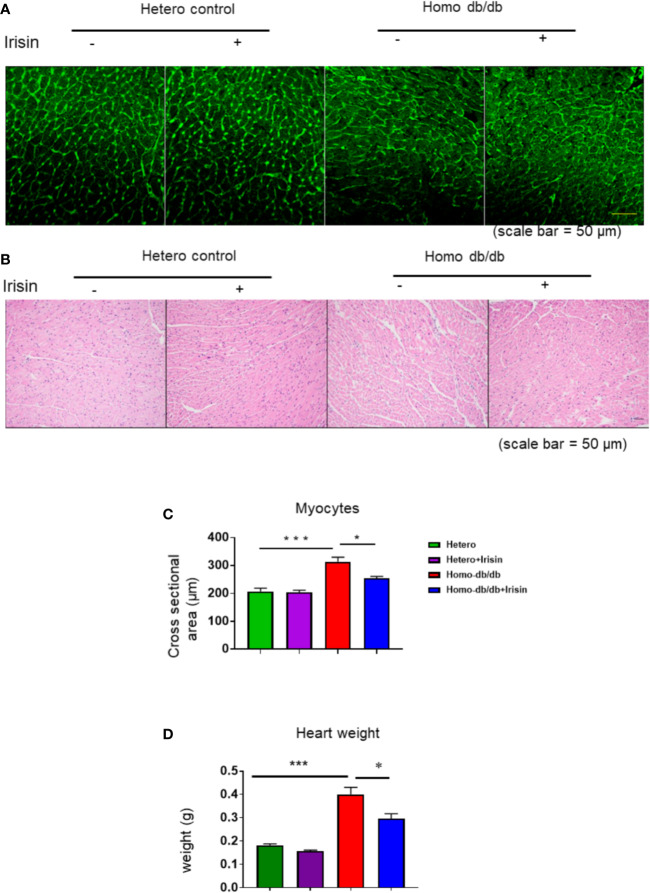
Effect of irisin attenuates the structural remodeling in homozygous db/db mice for spontaneous mutation (*Lepr^db^*) mice-. **(A, B)**. Representative images of wheat germ agglutinin (WGA) staining. **(C):** Quantitative analysis of myocyte cross-sectional area (heterozygous control=5; heterozygous+irisin=5; homozygous db/db control=5. homozygous db/db+irisin=4). **(D)**. The heart weight among different groups; Scale bar: 50 um. Data are shown as means ± SEM. *p < 0.05, ***p < 0.001.

**Figure 6:**
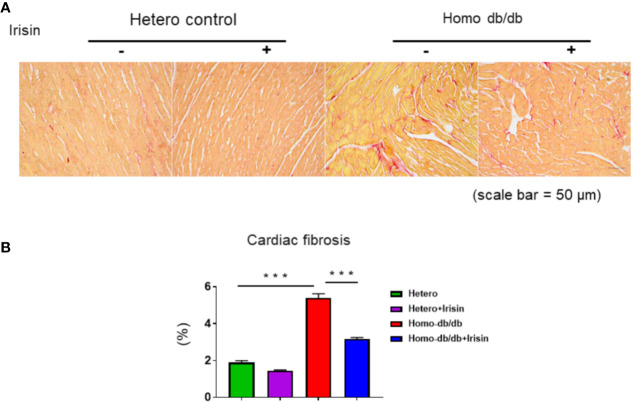
Irisin treatment suppressed interstitial fibrosis in the heart of homozygous db/db mice. **(A)**: Representative images of picrosirius red staining (scale bar: 50 um). **(B)**. Quantitative analysis of myocardial interstitial collagen deposition (heterozygous control=5; heterozygous+irisin=5; homozygous db/db control=5. homozygous db/db+irisin=5). Data are shown as means ± SEM. ***p < 0.001.

Homozygous db/db mice demonstrated an increased hypertrophic response as compared to heterozygous mice (0.182 ± 0.0068 in heterozygous control vs 0.400 ± 0.03 g in homozygous db/db control, p< 0.001). Treatment with irisin significantly reduced heart weight as compared to control treatment (0.297 ± 0.02 g in homozygous db/db +irisin vs 0.400 ± 0.03 in homozygous db/db control, p<0.05) (**Figures 5A, B**). Wheat germ agglutinin staining and H&E staining were employed to measure cardiomyocyte size. The homozygous db/db group had an increase in cross-sectional cardiomyocyte size versus heterozygous mice (207.09 ± 11.02 µm^2^ in heterozygous control vs 313.11 ± 16.53 µm^2^ in homozygous db/db control, p<0.001). However, the cross-sectional cardiomyocyte size and heart weight were reduced in homozygous db/db mice receiving irisin as compared with homozygous db/db mice alone (313.11 ± 16.53 µm^2^ in homozygous db/db control vs 253.88 ± 7.08 µm^2^, p<0.05) (**Figures 5C, D**). The collagen content was remarkedly augmented in myocardium from homozygous db/db mice as compared to heterozygous control mice (1.89% ± 0.09 in heterozygous control vs 5.39% ± 0.226 in homozygous db/db control, p<0.001) (**Figures 6A, B**). However, treatment of homozygous db/db mice with irisin lead to a significant suppression of interstitial collagen as compared with the vehicle control treatment of homozygous db/db mice (5.39% ± 0.226 in homozygous db/db control vs 3.17% ± 0.07 in homozygous db/db + irisin, p<0.001) (**Figures 6A, B**).

### Irisin Suppressed the Hypertrophic Response in Adipocytes in Homozygous Mice

**Figure 7:**
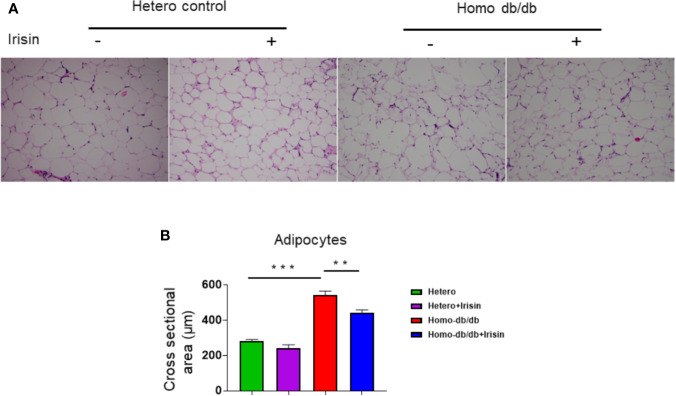
Irisin treatment suppressed hypertrophy of adipocytes in homozygous db/db mice. **(A)**: Representative images of HE staining; **(B)**: quantitative analysis of adipocyte sizes (heterozygous control=5; heterozygous+irisin=5; homozygous db/db control=5. homozygous db/db+irisin=5). Scale bar: 50 um. Values are shown as mean ± SEM, **p < 0.01, ***p < 0.001.

Adipocyte size was also estimated by H&E staining. As shown in **Figures 7A, B**, as compared to the heterozygous control group, homozygous db/db mice manifested an enlargement of adipocyte size (282.9 ± 8.34 µm^2^ in heterozygous control vs. 543.9 ± 21.2 µm^2^ in homozygous db/db control, p<0.001). However, treatment of db/db mice with irisin led to a remarkable reduction in adipocyte size as compared to the homozygous db/db mice vehicle treated group (543.9 ± 21.2 µm^2^ in homozygous db/db control vs 444.4 ± 14.8 µm^2^ in homozygous db/db +irisin, p<0.01), suggesting that irisin treatment resulted in a significant attenuation in the hypertrophic response in adipocytes.

### Irisin Treatments Activate Signaling Pathway

**Figure 8:**
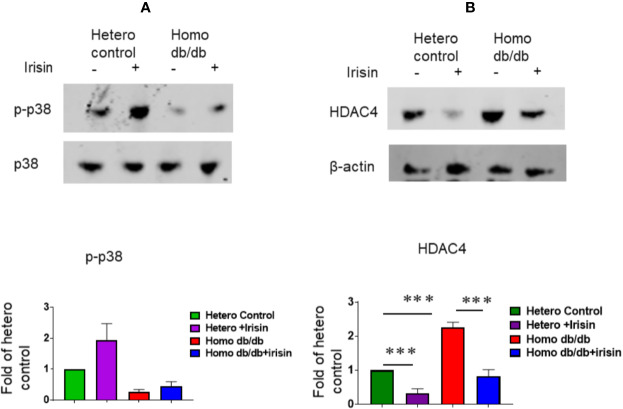
Irisin treatment increased p38 phosphorylation and attenuated the HDAC4 protein levels in the myocardium of homozygous db/db mice. **(A)**: The cardiac lysates were assessed by western blotting using anti-phosphorylated p38 and total p38 antibodies. Bar graph (lower panel) represents the densitometric analysis of phosphorylated p38, Values are shown as mean ± SEM (n = 3/per group). **(B)**: The cardiac lysates were assessed by western blotting using anti-HDAC4 and β-actin. Bar graph (lower panel) represents the densitometric analysis of HDAC4, Values are shown as mean ± SEM (heterozygous control=3; heterozygous+irisin=3; homozygous db/db control=3. homozygous db/db+irisin=3); ***p < 0.001.

We determine whether irisin treatment could induce the activation of p38, a downstream substrate of irisin. As demonstrated in **Figure 8A**, irisin treatment increased the phosphorylated p38 levels of myocardium from heterozygous mice. Homozygous myocardium showed decreased p38 phosphorylation as compared to the heterozygous heart. However, irisin treatment resulted in a marginal increase in p38 phosphorylation. In addition, it was reported that HDAC4 specifically responded to irisin in cardiomyoblasts exposed to hypoxia/reoxygenation. As shown in **Figure 8B**, the homozygous myocardium showed abundant HDAC4 proteins as compared to the heterozygous heart; treatment of mice with irisin caused the attenuation of HDAC4 in the myocardium in both heterozygous (p<0.001 vs heterozygous control) and homozygous mice (p<0.001 vs homozygous db/db control).

## Discussion

### Salient Findings and Perspectives

To the best of our acknowledge, this study is the first illustration that irisin protected the myocardium against cardiac dysfunction and by improving cardiac performance in homozygous db/db mice. Furthermore, irisin attenuated cardiac hypertrophy as indicated by the reduction of cardiomyocyte size and suppression of myocardial interstitial fibrosis in db/db mice. Likewise, irisin treatment mitigated hypertrophic responses in adipocytes homozygous db/db. Notably, the irisin treatment caused a remarkable improvement in insulin resistance as indicated by insulin tolerance test and glucose tolerance in association with the attenuation of hyperglycemia and hyperinsulinemia. We have also demonstrated that irisin-induced protective effect in homozygous db/db mice is related to p38 activation and reduction of HDAC4. Taken together, irisin preserved heart function, suppress cardiac remodeling, and attenuate metabolic disorders in homozygous db/db mice.

Irisin, as a recently identified hormone, is generated by the proteolytic processing of FNDC5 (Huh et al., 2012; Polyzos et al., 2014), abundantly existed in skeletal muscle, pericardium, heart, and brain (Wrann et al., 2013). The molecular basis of FNDC5 consists of a 209-residue protein with an N-terminal 29-residue signal sequence, which is followed by a putative fibronectin III (FNIII) 2 domain, a linking peptide, as well as a 39-residue cytoplasmic segment (Ferrer-Martínez et al., 2002; Raschke et al., 2013), which serves as a myokine by binding to a recently identified receptor (Schumacher et al., 2013).

Our recent studies indicated that treatment of mice with irisin reduced myocardial infarct size and increased post-ischemic cardiac performance (Wang et al., 2017), suggesting that irisin develops myocardial protection against ischemic injury. It was observed that irisin attenuated apoptotic proteins in ischemic heart, which also suggest that anti-apoptosis plays an important role for irisin-induced protective effect, which was also associated with increased anti-oxidant function (Tamura et al., 1988; Wang et al., 2017) In this observation, we have found that irisin treatment rescued homozygous db/db mice from developing a severer myocardial dysfunction as compared to control treatment. The improved myocardial function induced by irisin was illustrated in homozygous db/db heart, however, specific signaling pathway responsible for functional improvements homozygous db/db myocardium remains determined following the administration of irisin. It has been demonstrated that systemic insulin resistance and metabolic disorders accelerated the progression of cardiac dysfunction in the development of heart failure (Zhang et al., 2017). Consistent with these observations, our study indicates that irisin treatment significantly improved systemic insulin resistance and mitigated the extent of hyperglycemia and hyperinsulinemia, which is associated with cardiac dysfunction. There are several experimental diabetic models for studying the mechanism(s) and pathological phenotype associated with diabetes, but each model displays certain limitations, and no perfect model could phenocopy the condition of diabetes in humans. We used the high fat diet (HFD)-diet induced type II diabetic model in our previous study, which has a unique advantage for studying diet induced obesity, insulin resistance, and diabetes. Relative to the diet induced diabetic model, we included db/db mice that are deficient in the leptin receptor. This model is characterized by the development of severe type 2 diabetes very rapidly in association with early hyperinsulinemia, metabolic disorder, and pronounced myocardial disfunction (Bugger and Abel, 2009). Although the current study demonstrates the marked protective effects of irisin on a db/db diabetic model, it is not clear whether irisin could manifest a different function and phenotype between db/db mice and high fat diet-induced type II diabetic models. We have found that irisin could induce the protection in myoblasts in association with histone deacetylase (Zhao et al., 2016). Our previous works indicated that HDAC inhibition resulted in cardioprotective effects against cellular and cardiac ischemic and reperfusion injuries and hypertrophic response (Gallo et al., 2008; Zhang et al., 2012; Du et al., 2015). Very recently, targeting HDAC has been demonstrated as a promising approach to exploring a new therapy for diabetes (Newman and Verdin, 2014; Lundh et al., 2015). Inhibition of HDACs has been demonstrated to increase insulin secretion and prevent metabolic disorders (Lundh et al., 2015). We have shown that irisin-induced cardiac repair and angiogenesis following cell engraftment into damaged heart, which is closely related to the upregulation of acetylation and attenuation of histone deacetylase activity. It is likely that irisin-induced cardiac protection in homozygous db/db mice was due to the inhibition of HDAC pathway, which merits the further investigation in our future studies.

Evidence has suggested the functional role of irisin on the body metabolism and thermogenesis (Spiegelman, 2013; Rachid et al., 2015). The beneficial effects of irisin on metabolic disorders are associated with not only the driving of the browning of white adipose tissue, increasing the energy expenditure, but also suppressing inflammatory response and oxidative stress (Polyzos et al., 2014; Dulian et al., 2015; Lu et al., 2015; Zhu et al., 2015). This is in agreement with our observation that insulin intolerance was promoted in homozygous db/db mice during the course of progression into the heart dysfunction, which was prevented by the administration of irisin as compared to the control treatment. We have also found that HDAC inhibition mitigated insulin resistance in the HFD-induced diabetes model in our previous observation (Zhang et al., 2017). It is not clear whether improvement in insulin sensitivity in homozygous db/db mice shares a similar mechanism that occurred in HFD model, or hypertrophic response in muscles and adipocytes in homozygous db/db, which was suppressed by irisin treatment as compared to control treatment. We have previously found that HDAC4 was attenuated following irisin treatments in cardiomyoblasts exposed to hypoxia/reoxygenation (Zhao et al., 2016). In agreement with observation, this study showed that abundant HDAC4 protein exhibited in the myocardium of homozygous db/db mice, but irisin treatment caused a dramatic decrease in HDAC4 in the myocardium, indicating that suppression of HDAC4 might be associated with the protective effect of irisin against cardiac dysfunction in homozygous db/db mice. It is interesting in the future to employ cultured cells to examine whether the manipulation of HDAC4 activity could also be involved in irisin-induced cellular protection. Furthermore, p38 activation was considered to be important pathway by which irisin execute to achieve biological function (Zhang et al., 2014). Our finding indicates that p38 phosphorylation was attenuated in myocardium of homozygous mice, but irisin led to the increase in p38 phosphorylation, suggesting that p38 phosphorylation is involved in irisin-induced protective effects.

In conclusion, this study has demonstrated that irisin preserves cardiac performance of homozygous db/db mice as compared to vehicle treatment, as indicated by improving cardiac function during the development of cardiac dysfunction. In addition, myocardial protection of irisin in homozygous db/db mice was closely related to attenuation of cardiac hypertrophy and interstitial fibrosis. Notably, insulin resistance, hyperglycemia, and hyperinsulinemia as demonstrated in homozygous db/db mice were significantly suppressed by administration of irisin. It is also demonstrated that irisin-induced the protective effect is related to activation of p38 and reduction of HDAC4. Taken together, our study illustrates that irisin serves as a novel approach to inducing protective effect against myocardial dysfunction and insulin resistance in genetic diabetic model, which holds promise in developing a new therapeutic strategy in cardiac protection, diabetes, and obesity.

## Data Availability Statement

All datasets generated for this study are included in the article/supplementary material.

## Ethics Statement

The animal study was reviewed and approved by Roger William Medical Center.

## Author Contributions

JW, YZ, and LZ performed experiments and statistical analysis about ventricular function, metabolic measurement, and western blot. TZ, PD, YC, and GQ designed experiment. TZ, SZha, and SZhu wrote and edited manuscript.

## Funding

The study is supported by the National Institutes of Health (NHLBI) (R01 HL089405 and R01 HL115265).

## Conflict of Interest

The authors declare that the research was conducted in the absence of any commercial or financial relationships that could be construed as a potential conflict of interest.
